# Discovery and replication of a peripheral tissue DNA methylation biosignature to augment a suicide prediction model

**DOI:** 10.1186/s13148-016-0279-1

**Published:** 2016-11-03

**Authors:** Makena L. Clive, Marco P. Boks, Christiaan H. Vinkers, Lauren M. Osborne, Jennifer L. Payne, Kerry J. Ressler, Alicia K. Smith, Holly C. Wilcox, Zachary Kaminsky

**Affiliations:** 1 Department of Psychiatry and Behavioral Sciences, Johns Hopkins University School of Medicine, Baltimore, MD 21205 USA; 2 Department of Psychiatry, Brain Center Rudolf Magnus, University Medical Center Utrecht (UMCU), Utrecht, The Netherlands; 3 Department of Psychiatry, McLean Hospital, Harvard Medical School, Boston, MA 02478 USA; 4 Department of Psychiatry and Behavioral Sciences, Emory University School of Medicine, Atlanta, GA 30322 USA; 5 Howard Hughes Medical Institute, Chevy Chase, MD USA; 6 Department of Mental Health, Johns Hopkins Bloomberg School of Public Health, Baltimore, MD 21218 USA; 7 The Mood Disorder Center, Johns Hopkins University, 720 Rutland Avenue, Ross Research Building 1070, Baltimore, MD 21205 USA

**Keywords:** Suicide, Biomarker, Epigenetics, DNA methylation, SKA2, HPA axis, Illumina HM450 microarray, Childhood trauma

## Abstract

**Background:**

Suicide is the second leading cause of death among adolescents in the USA, and rates are rising. Methods to identify individuals at risk are essential for implementing prevention strategies, and the development of a biomarker can potentially improve prediction of suicidal behaviors. Prediction of our previously reported *SKA2* biomarker for suicide and PTSD is substantially improved by questionnaires assessing perceived stress or anxiety and is therefore reliant on psychological assessment. However, such stress-related states may also leave a biosignature that could equally improve suicide prediction. In genome-wide DNA methylation data, we observed significant overlap between waking cortisol-associated and suicide-associated DNA methylation in blood and the brain, respectively.

**Results:**

Using a custom bioinformatic brain to blood discovery algorithm, we derived a DNA methylation biosignature that interacts with *SKA2* methylation to improve the prediction of suicidal ideation in our existing suicide prediction model across both blood and saliva data sets. This biosignature was independently validated in the Grady Trauma Project cohort and interacted with HPA axis metrics in the same cohort. The biosignature showed a relationship with immune status by its correlation with myeloid-derived cell proportions in all data sets and with IL-6 measures in a prospective postpartum depression cohort. Three probes showed significant correlations with the biosignature: cg08469255 (*DDR1*), cg22029879 (*ARHGEF10*), and cg24437859 (*SHP1*), of which *SHP1* methylation correlated with immune measures.

**Conclusions:**

We conclude that this biosignature interacts with *SKA2* methylation to improve suicide prediction and may represent a biological state of immune and HPA axis modulation that mediates suicidal behavior.

**Electronic supplementary material:**

The online version of this article (doi:10.1186/s13148-016-0279-1) contains supplementary material, which is available to authorized users.

## Background

Suicide accounts for 1.4 % of worldwide deaths annually, posing a serious public health issue [1]. Based on 2014 data, it is the second leading cause of death among adolescents, and the tenth leading cause of death for all ages in the USA [2]. Given the rising rates of suicide in the USA, methods to identify individuals at risk for implementing prevention strategies are urgently needed [3].

Recently, our laboratory identified a DNA methylation mark that is associated with suicide in a postmortem brain tissue cohort at a CpG (cg13989295) located within a single nucleotide polymorphism (SNP), rs7208505, in the spindle- and kinetochore-associated protein 2 (*SKA2*) where the reference allele of rs7208505 eliminates the CpG. The observed epigenetic association with suicide was replicated in two additional brain tissue cohorts and with suicidal behaviors including suicidal ideation (SI) and attempt (SA) in peripheral blood in three living cohorts [4]. In our original work, gene expression of *SKA2* was correlated with DNA methylation at this position and was significantly decreased in suicide decedents. Several recent independent studies have observed decreased expression of *SKA2* in both the blood of violent suicide completers [5] and in the prefrontal cortex of suicide victims [4, 6], the latter of which was also associated with decreased protein levels.

The SKA2 protein is thought to interact with the hypothalamic-pituitary-adrenal (HPA) axis by chaperoning the glucocorticoid receptor (GR) from the cytoplasm to the nucleus upon cortisol binding [7]. Once in the nucleus, the GR can interact with genomic DNA and influence gene expression involved in negative feedback regulation of the HPA axis response. In two independent cohorts with high levels of childhood trauma, elevated *SKA2* DNA methylation in peripheral blood before administration of the TRIER social stress test was significantly associated with a blunted post-test cortisol level, while SKA2 DNA methylation before the dexamethasone suppression test (DST) was significantly associated with elevated post-test cortisol levels [8, 9]. These data support the interpretation that *SKA2* DNA methylation state may be an important contributor to individual stress response.

In an attempt to identify at-risk individuals, we previously generated a suicide prediction model, which describes suicidal behavior as a function of both genotype and methylation at the single nucleotide polymorphism (SNP) rs7208505 in *SKA2* which interacts with a state level metric of stress or trait level metric of anxiety to confer risk [4]. Notably, some studies demonstrate that state level stress can be influenced by trait level anxiety [10]. Model predictive accuracies vary between ~70 and 85 % in various cohorts and are consistent with *SKA2* gene expression-based prediction accuracies reported by other groups [8, 11]. The statistical interaction with stress is likely related to the physiological role *SKA2* plays in mediating HPA axis activity. In this context, it is hypothesized that epigenetic variation of *SKA2* may represent an underlying trait vulnerability of the HPA axis that must interact with a state of stress to elicit risk. In our previous work, we have identified significant interactions of *SKA2* with various self-reported psychological scales to influence suicide risk. The scales vary by study cohort and include the Child Trauma Questionnaire (CTQ), the Perceived Stress Scale, waking salivary cortisol levels, and various metrics of anxiety including self-reported binary estimates and those quantified by the Self-Report for Childhood Anxiety Related Disorders (SCARED) [4, 8, 9]. Furthermore, our work and others have noted an increased model efficacy in subgroups of individuals having experienced childhood trauma [8, 9, 12, 13]. It is possible that high values in the stress metrics represent a biological state that may be related to HPA axis function. Despite the possibility to assess these states using questionnaires, the use of self-reported scales has many drawbacks including a lack of standardization across studies, variability in psychometric properties, and variability in the subjective rating of stress levels. In the clinical context, the administration of questionnaires requires time and patient compliance.

Recent attempts have been made to circumvent the use of psychological assessments and develop biomarker proxies [11, 14]. A challenge for the identification of peripheral tissue-based epigenetic biomarkers in the context of psychiatry is the generalizability of the identified peripheral epigenetic variation in the brain. We have hypothesized previously that circulating steroid hormones such as cortisol may mark peripheral tissue DNA on the epigenetic level while affecting behavior through central nervous system (CNS) specific actions [15–17]. In support of this hypothesis, the initial objective of this study was to evaluate if cortisol-associated DNA methylation levels in peripheral tissues (blood and saliva) are enriched among suicide-associated DNA methylation levels in the brain.

While systemic factors like cortisol may influence epigenetic patterns across-tissues and may represent relevant biomarkers interacting with SKA2, we did not wish to limit our analysis to cortisol-associated probes alone. Thus, the second major objective of this study was to generate an epigenetic biosignature of *SKA2-*interacting state markers in a bioinformatically driven and unbiased manner and to understand the underlying biological context driving any identified biosignatures.

## Results

### Overrepresentation of peripheral cortisol-associated loci among brain-associated suicide genes

To address our first objective, we attempted to address the degree to which peripheral blood- or saliva-based DNA methylation profiles are indicative of epigenetic profiles in the brain related to suicidal behaviors. One potential substrate for peripheral tissue-brain overlap is a cross-tissue reprogramming by the systemic influence of hormones. For Genetics of Recurrent Early-Onset Depression (GenRED) Offspring, we identified 20,146 and 22,865 probes that were nominally associated with the area under the curve (AUC) of waking weekday cortisol in blood and saliva samples, respectively (Additional files 1 and 2). To increase statistical power, we performed a combined analysis of blood and saliva samples using a linear model with age, sex, tissue type (blood or saliva), and cell type proportion as covariates and identified 22,425 loci associated with the AUC of waking weekday cortisol (Additional file 3). A pathway enrichment analysis of the genes significantly associated (*P* < 0.001) with the AUC of weekday waking cortisol in GenRED Offspring blood and saliva using the tool g:Profiler revealed an enrichment of neural development pathways at varying levels of significance (Table 1) [18, 19]. Given the importance of dysregulated cortisol biology to suicidal behaviors, cortisol-associated methylation probes in peripheral blood (*N* = 18) and saliva (*N* = 20) from the GenRED Offspring cohorts were assessed for an overrepresentation with those probes significantly associated with completed suicide separately in postmortem prefrontal cortical neurons and non-neurons (*N* = 45). Cortisol-associated probes within genes or gene regulatory sites were significantly overrepresented among prefrontal neuron suicide-associated genes and genes previously identified as associated with cortisol stress reactivity (Table 2) [20]. These findings indicate that there may be common pathways between cortisol biology and suicidal behavior and that the epigenetic marks of suicide-associated hormonal changes may be detectable in peripheral tissues.

**Table 1:** Gene ontology results

Rank	Probability estimate	Expected probability	*P* value	Term ID	Term name
1	0.211	0.053	1.74 × 10^−5^	GO:0007399	Nervous system development
2	0.15	0.059	3.63 × 10^−5^	GO:0030182	Neuron differentiation
3	0.156	0.057	6.40 × 10^−5^	GO:0048699	Generation of neurons
4	0.161	0.056	8.19 × 10^−5^	GO:0022008	Neurogenesis
5	0.128	0.061	1.42 × 10^−4^	GO:0048666	Neuron development
6	0.809	0.036	2.94 × 10^−4^	GO:0044424	Intracellular part
7	0.111	0.063	3.77 × 10^−4^	GO:0000904	Cell morphogenesis involved in differentiation
8	0.093	0.066	9.76 × 10^−4^	GO:0048667	Cell morphogenesis involved in neuron differentiation
9	0.224	0.048	1.08 × 10^−3^	GO:0009653	Anatomical structure morphogenesis
10	0.085	0.067	2.16 × 10^−3^	GO:0007409	Axonogenesis
11	0.184	0.05	3.32 × 10^−3^	GO:0048468	Cell development
12	0.093	0.064	3.37 × 10^−3^	GO:0048812	Neuron projection morphogenesis
13	0.271	0.045	3.62 × 10^−3^	GO:0065008	Regulation of biological quality
14	0.817	0.035	5.78 × 10^−3^	GO:0005622	Intracellular
15	0.271	0.045	6.13 × 10^−3^	GO:0009893	Positive regulation of metabolic process
16	0.085	0.065	6.92 × 10^−3^	GO:0061564	Axon development
17	0.362	0.041	0.0105	GO:0048856	Anatomical structure development
18	0.709	0.036	0.0107	GO:0043229	Intracellular organelle
19	0.108	0.058	0.0112	GO:0031175	Neuron projection development
20	0.279	0.044	0.0161	GO:0030154	Cell differentiation

Gene ontology analysis of genes significantly associated with weekday AUC cortisol measured in GenRED Offspring blood and saliva samples using the tool g:Profiler

**Table 2:** Overrepresentation analysis

Cohort	Comparison	Significance level	Expected probability	Probability estimate	Binomial test *P* value
GenRED Offspring Saliva, cortisol	Neuron, suicide	0.05	0.507	0.614	3.47 × 10^−102^
		0.01	0.158	0.242	8.70 × 10^−28^
		0.001	0.019	0.026	0.332
GenRED Offspring Blood, cortisol	Neuron, suicide	0.05	0.470	0.569	2.97 × 10^−86^
		0.01	0.132	0.209	1.30 × 10^−26^
		0.001	0.015	0.039	9.71 × 10^−3^
Combined GenRED Offspring Blood and saliva, cortisol	Neuron, suicide	0.05	0.522	0.618	1.68 × 10^−81^
		0.01	0.187	0.251	1.34 × 10^−15^
		0.001	0.032	0.035	0.851
Houtepen et al*.* [20] Cortisol	Neuron, suicide	0.05	0.470	0.578	2.01 × 10^−105^
		0.01	0.119	0.193	6.02 × 10^−32^
		0.001	0.011	0.029	3.15 × 10^−3^
Combined GenRED Offspring Blood and saliva, cortisol	Houtepen et al*.* [20] Cortisol	0.05	0.522	0.603	6.70 × 10^−60^
		0.01	0.187	0.264	1.08 × 10^−25^
		0.001	0.032	0.061	4.96 × 10^−3^

Overrepresentation analysis of genes significantly associated with AUC weekday cortisol measures in GenRED Offspring blood and saliva samples, genes significantly associated with suicide in postmortem prefrontal cortex neurons, and genes associated with AUC cortisol in blood from Houtepen et al*.* [20]

### Algorithmic identification of SKA2-interacting biosignature for DNA methylation-based suicidal behavior prediction

**Fig. 1:**
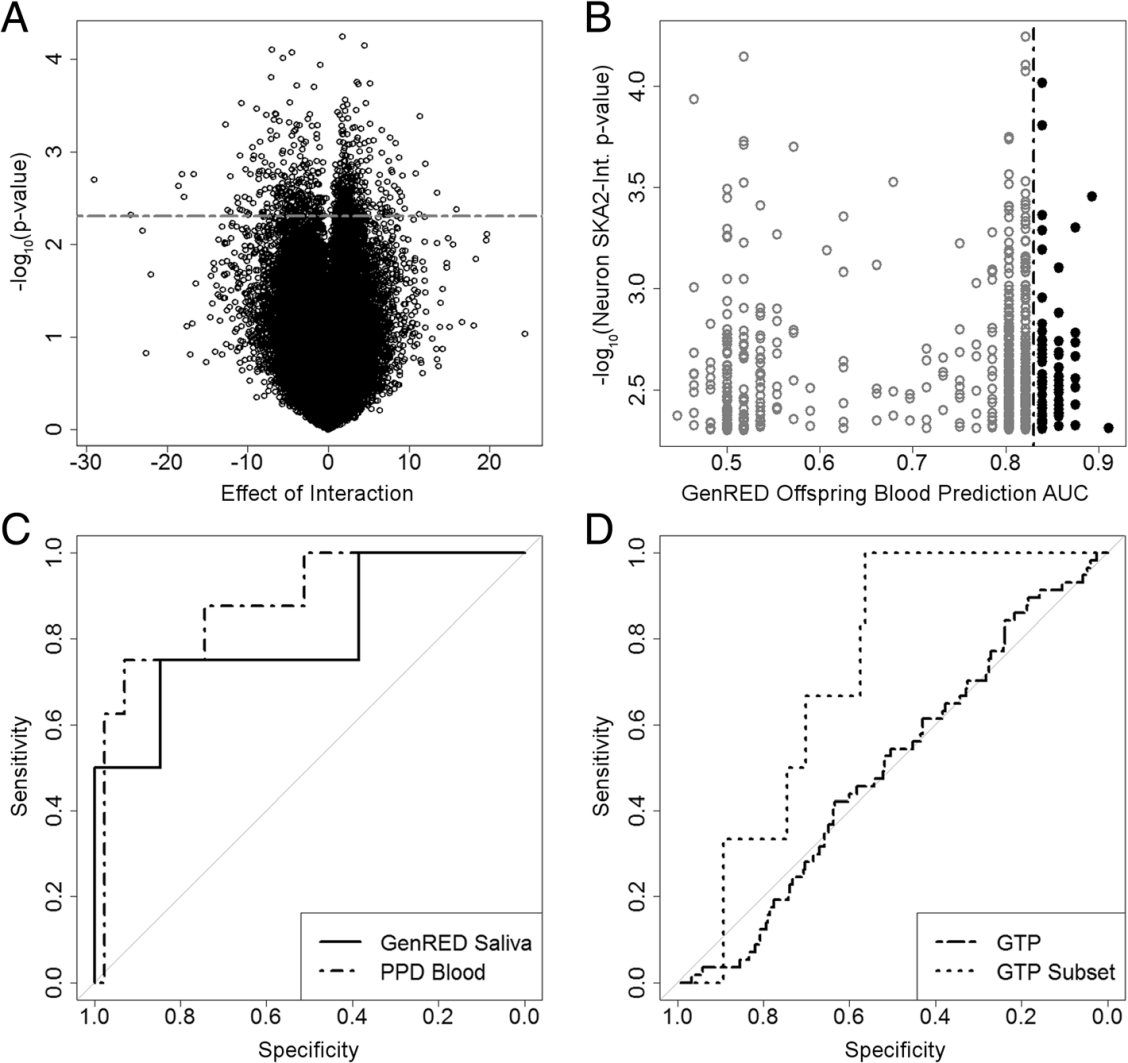
Discovery of interaction biosignature probes and prediction of suicidal ideation using interaction biosignature in multiple cohorts. **a** Volcano plot in prefrontal cortex neurons (cases, *N* = 22; controls, *N* = 23) of the interaction of individual probe methylation with rs7208505 methylation and genotype. **b** Probes with an interaction *P* value <0.005 (*N* = 669) were optimized for prediction of SA in GenRED Offspring blood. Probes with an AUC prediction above 0.825 (*N* = 72) were used to train a PCA model. **c** ROC curves of prediction of SI in GenRED Offspring saliva and PPD cohort blood using the first eigenvectors of predicted PCAs. **d** ROC curves of prediction of SI in the whole GTP cohort and a subset of drug-naïve, non-PTSD individuals (cases = 6; controls = 109)

In light of the above findings, our strategy to approach our second objective of generating a biosignature of *SKA2-*interacting state markers was to identify epigenetic variation interacting with *SKA2* that was consistent across brain and peripheral tissues. The full algorithm is explained in Additional file 4: Figure S1. Briefly, DNA methylation in prefrontal cortex neurons at each probe was assessed for statistical interaction with rs7208505 CpG methylation (chr17:59110368, hg38) in a linear model controlling for age, sex, and rs7208505 genotype and identified 669 probes below a *P* value cutoff of 0.005 (Fig. 1a, Additional file 4: Result S1). Of these 669 probes, 72 exhibited an AUC prediction for SA in the top 25th percentile (AUC > 0.825) in the GenRED Offspring blood cohort (Fig. 1b; Table 3). The methylation at these 72 probes was used to train a principal component analysis (PCA) model on the GenRED Offspring blood data, which was then used to predict PCA models in the other data sets. The first eigenvector was used to assess suicidal behavior prediction in the original prediction model, replacing the stress measure with the PCA first eigenvector. All steps were evaluated by the Monte Carlo method and found to be statistically significant (*P* < 0.001, Additional file 4: Result S1). This approach predicted SI in PPD cohort blood with an AUC of 0.88 (95 % CI 0.75–1; *P* = 0.041) and in GenRED Offspring saliva with an AUC of 0.81 (95 % CI 0.59–1; *P* = 0.011) (Fig. 1c; Table 4). These high-prediction AUCs provide evidence that the PCA first eigenvector may represent a methylation *SKA2* “interaction biosignature” that is predictive of suicidal behavior in the existing suicide prediction model and replaces the need for a stress questionnaire.

**Table 3:** Probes from interaction biosignature contributing to the PCA model

Probe ID	Location (hg19)	Gene symbol	Gene name
cg01060471	chr10:103911733	NOLC1	Nucleolar and coiled body phosphoprotein 1
cg01252219	chr12:110302105	GLTP	Glycolipid transfer protein
cg02068690	chr2:25600451	DTNB	Dystrobrevin, beta
cg02097235	chr16:1116799	SSTR5-AS1	SSTR5 antisense RNA 1
cg02246725	chr11:2014127	HOTS	H19 opposite tumor suppressor
cg02340818	chr8:145808436	ARHGAP39	Rho GTPase activating protein 39
cg02464608	chr3:122631723	SEMA5B	Semaphorin 5B
cg02516957	chr8:128011063		
cg02953125	chr2:1079100	SNTG2	Syntrophin, gamma 2
cg03198117	chrX:152939967	PNCK	Pregnancy up-regulated nonubiquitous CaM kinase
cg03351894	chrX:48686200	ERAS	ES cell expressed Ras
cg03782771	chr4:152801238		
cg03887528	chr2:231090531	SP140	SP140 nuclear body protein
cg06363485	chr6:41207376	TREML4	Triggering receptor expressed on myeloid cells like 4
cg06774087	chr5:79647614	CRSP8P	Mediator complex subunit 27 pseudogene
cg07483709	chr10:29439573		
cg07589300	chr4:1404128		
cg07787977	chr1:962651	AGRN	Agrin
cg08119631	chrX:118822815	SEPT6	Septin 6
cg08469255	chr6:30851069	DDR1	Discoidin domain receptor tyrosine kinase 1
cg08674415	chr16:34430905		
cg08720806	chr11:125142671	PKNOX2	PBX/knotted 1 homeobox 2
cg08795752	chr9:136293271	ADAMTS13	ADAM metallopeptidase with thrombospondin type 1 motif, 13
cg09105334	chr17:15683060		
cg11450546	chr19:43965012	LYPD3	LY6/PLAUR domain containing 3
cg11496113	chr5:34627766		
cg11780096	chr2:178976254	RBM45	RNA binding motif protein 45
cg11880367	chr10:80141181	LINC00856	
cg12378817	chr10:133961235	JAKMIP3	Janus kinase and microtubule interacting protein 3
cg12622680	chr7:158819620	LINC00689	
cg12833118	chr4:2572546		
cg12967319	chr14:101291997	MEG3	Maternally expressed 3 (non-protein coding)
cg13466253	chr20:3731334	HSPA12B	Heat shock 70kD protein 12B
cg13798679	chr1:36617570	TRAPPC3	Trafficking protein particle complex 3
cg14439102	chr9:80360719	GNAQ	Guanine nucleotide binding protein (G protein), q polypeptide
cg14509196	chr15:25494565	SNORD115-44	Small nucleolar RNA, C/D box 115-44
cg15071166	chr17:3771325	CAMKK1	Calcium/calmodulin-dependent protein kinase kinase 1, alpha
cg15302379	chr10:102821848	KAZALD1	Kazal-type serine peptidase inhibitor domain 1
cg15508401	chr2:239997380	HDAC4	Histone deacetylase 4
cg15677087	chr20:61584850	SLC17A9	Solute carrier family 17, member 9
cg15838142	chr17:77184010	RBFOX3	RNA binding protein, fox-1 homolog 3
cg16900102	chr7:98283412		
cg16943635	chr11:62067691	SCGB1D4	Secretoglobin, family 1D, member 4
cg17267737	chr7:158819531	LINC00689	
cg17813840	chr15:79852956		
cg18144654	chr3:127995346	EEFSEC	Eukaryotic elongation factor, selenocysteine-tRNA-specific
cg18546110	chr4:10763497		
cg19688321	chrX:70476177	BCYRN1	Brain cytoplasmic RNA 1
cg20704602	chr6:29635371	MOG	Myelin oligodendrocyte glycoprotein
cg20929545	chr11:118958046	HMBS	Hydroxymethylbilane synthase
cg21066636	chr17:4675292	TM4SF5	Transmembrane 4 L six family member 5
cg21352158	chr8:832917	ERICH1-AS1	ERICH1 antisense RNA 1
cg21407196	chr1:46751975	LRRC41	Leucine rich repeat containing 41
cg21729235	chrX:100739272	ARMCX4	Armadillo repeat containing, X-linked 4
cg22029879	chr8:1790861	ARHGEF10	Rho guanine nucleotide exchange factor 10
cg22133973	chr6:170789640	PSMB1	Proteasome subunit beta 1
cg22184136	chr6:30038720	RNF39	Ring finger protein 39
cg22939113	chr19:40719949	MAP3K10	Mitogen-activated protein kinase kinase kinase 10
cg22954621	chr10:13514626	BEND7	BEN domain containing 7
cg23043139	chr3:13678918	FBLN2	Fibulin 2
cg23374863	chr14:94984140	SERPINA12	Serpin family A member 12
cg24112628	chr6:150174215	LRP11	Low-density lipoprotein receptor-related protein 11
cg24416190	chr4:186007379		
cg24437859	chr12:7066614	PTPN6	Protein tyrosine phosphatase, non-receptor type 6
cg25132241	chr14:92396859	FBLN5	Fibulin 5
cg25133951	chr1:178575267		
cg25215117	chr17:11461665	SHISA6	Shisa family member 6
cg25447359	chr22:30790057		
cg25477843	chr8:145061318	PARP10	Poly (ADP-ribose) polymerase family, member 10
cg26224354	chr7:1096374	GPR146	G protein-coupled receptor 146
cg26305504	chr19:947612	ARID3A	AT-rich interactive domain 3A
cg26493346	chr16:1812273	MAPK8IP3	Mitogen-activated protein kinase 8 interacting protein 3

**Table 4:** Prediction model results

Cohort	N	Outcome	Cases	Controls	Interaction	AUC	95 % CI	*P* value
GenRED Offspring blood	18	Attempt	4	14	Biosignature	0.768	0.47–1	0.052
GenRED Offspring saliva	20	Ideation	5	15	Biosignature	0.807	0.59–1	0.011
PPD cohort blood	51	Ideation	8	43	Biosignature	0.884	0.75–1	0.041
GTP blood	376	Ideation	63	313	Biosignature	0.500	0.42–0.58	0.724
GTP subset blood	115	Ideation	6	109	Biosignature	0.727	0.59–0.87	0.050
GenRED Offspring blood	18	Attempt	4	14	% myeloid	0.732	0.40–1	0.28
GenRED Offspring saliva	20	Ideation	5	15	% myeloid	0.788	0.56–1	0.28
PPD cohort blood	51	Ideation	8	43	% myeloid	0.834	0.61–1	0.003
GTP subset blood	115	Ideation	6	109	% myeloid	0.728	0.55–0.9	0.99

Prediction model results for all cohorts using either the interaction biosignature or the myeloid-derived proportion as the interaction term. *P* values are derived from Monte Carlo permutation

### Independent validation of SKA2 model interaction biosignature performance

The interaction biosignature model was validated using methylation array data from the Grady Trauma Project (GTP), a sample of urban minorities with low socioeconomic status and high rates of traumatic experience and PTSD. On the entire sample, the prediction model generated an AUC of 0.50 (95 % CI 0.42–0.58; *P* = 0.724) for prediction of SI in all 376 individuals. Based on recent publications that have provided evidence that both PTSD and substance abuse may confound *SKA2* methylation [9, 21], we selected a subset of the GTP sample with no history of PTSD or drug use (*N* = 115; 6 cases, 109 controls), where a combination of *SKA2* and the interaction biosignature predicted SI with an AUC of 0.73 (95 % CI 0.59–0.87; *P* = 0.050) (Fig. 1d; Table 4) [12]. Although our interaction biosignature model was unsuccessful in suicidal behavior prediction across the entire GTP cohort, prediction was successful in a subset without PTSD. This altered suicidal behavior prediction with PTSD is consistent with previously published findings [9].

### Association of interaction biosignature metrics with HPA axis function

To improve our understanding of the biological relevance of the interaction biosignature, we assessed biosignature loci for a relationship with various metrics of HPA axis function in two cohorts with high levels of childhood trauma as assessed by the CTQ. The interaction biosignature eigenvector interacted with CTQ scores to associate with post-test AUC cortisol levels following the administration of the TRIER social stress test (biosignature β = 3446.9 ± 1631.2, *P* = 0.038; CTQ β = −40.6 ± 12.9, *P* = 0.002; interaction β = −92.8 ± 45.0, *P* = 0.043, F = 4.5, df = 4/81, model *P* = 0.038) (Additional file 4: Figure S2A). In the GTP sample subset, the interaction biosignature eigenvector interacted with CTQ scores to associate with the natural log of the day 2 cortisol following administration of the DST (biosignature β = −6.4 ± 2.8, *P* = 0.027; CTQ β = 0.096 ± 0.037, *P* = 0.012; interaction β = 0.22 ± 0.095, *P* = 0.027, F = 2.4, df = 4/49, model *P* = 0.027) (Additional file 4: Figure S2B). Taken together, the data suggest that *SKA2* interaction biosignature values associate with early life trauma status to influence HPA axis sensitivity.

### Assessment of biological relevance of SKA2 model interaction biosignature

**Fig. 2:**
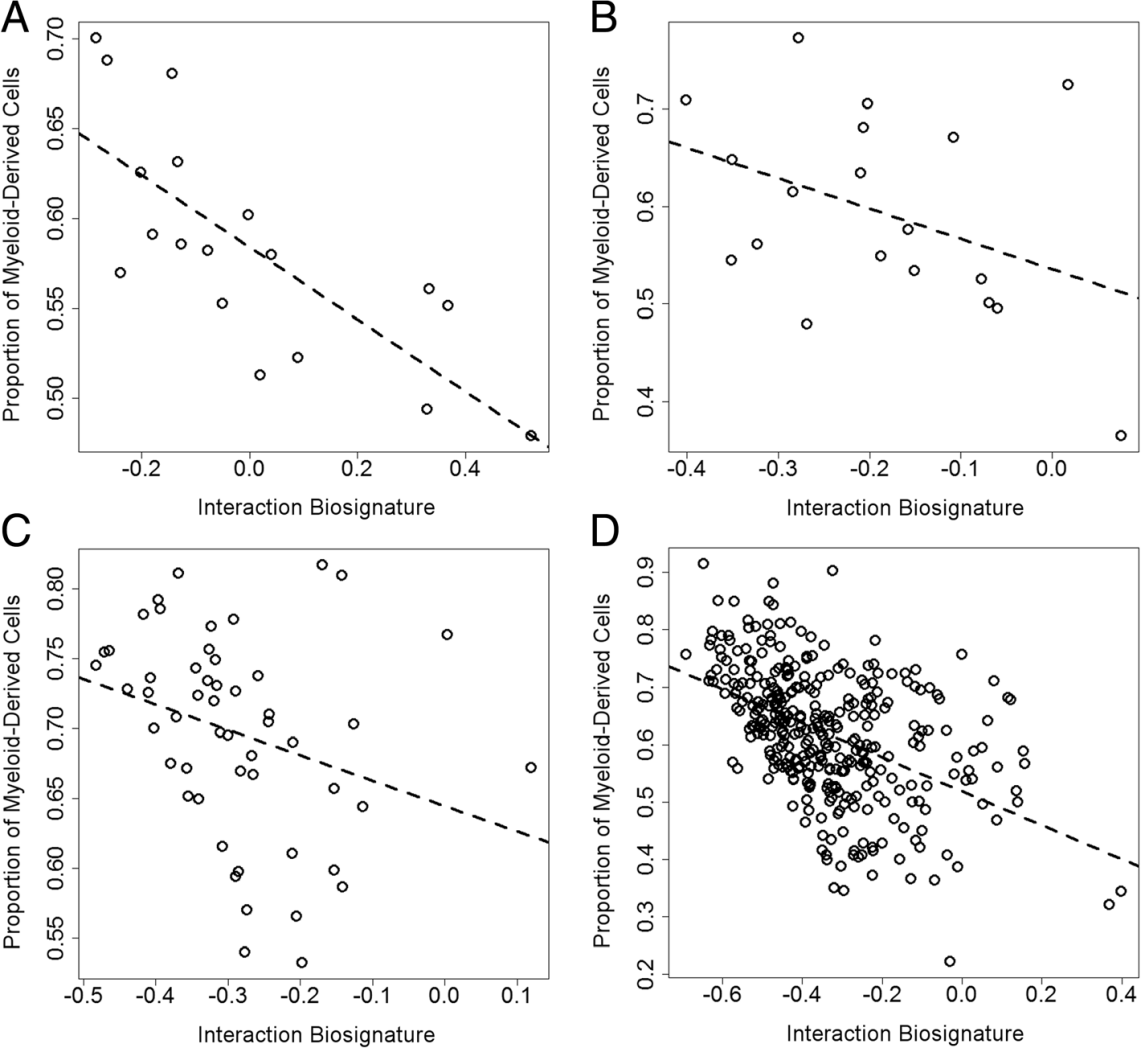
Myeloid-derived cell proportions correlated with the interaction biosignature in all cohorts and were predictive of suicidal behavior. Correlations were observed between the interaction biosignature and the proportion of myeloid-derived cells in **a** GenRED Offspring blood (*P* = 2.7 × 10^−4^), **b** saliva (*P* = 0.092), **c** PPD cohort blood (*P* = 0.034), and **d** GTP blood (*P* = 2.4 × 10^−7^)

**Fig. 3:**
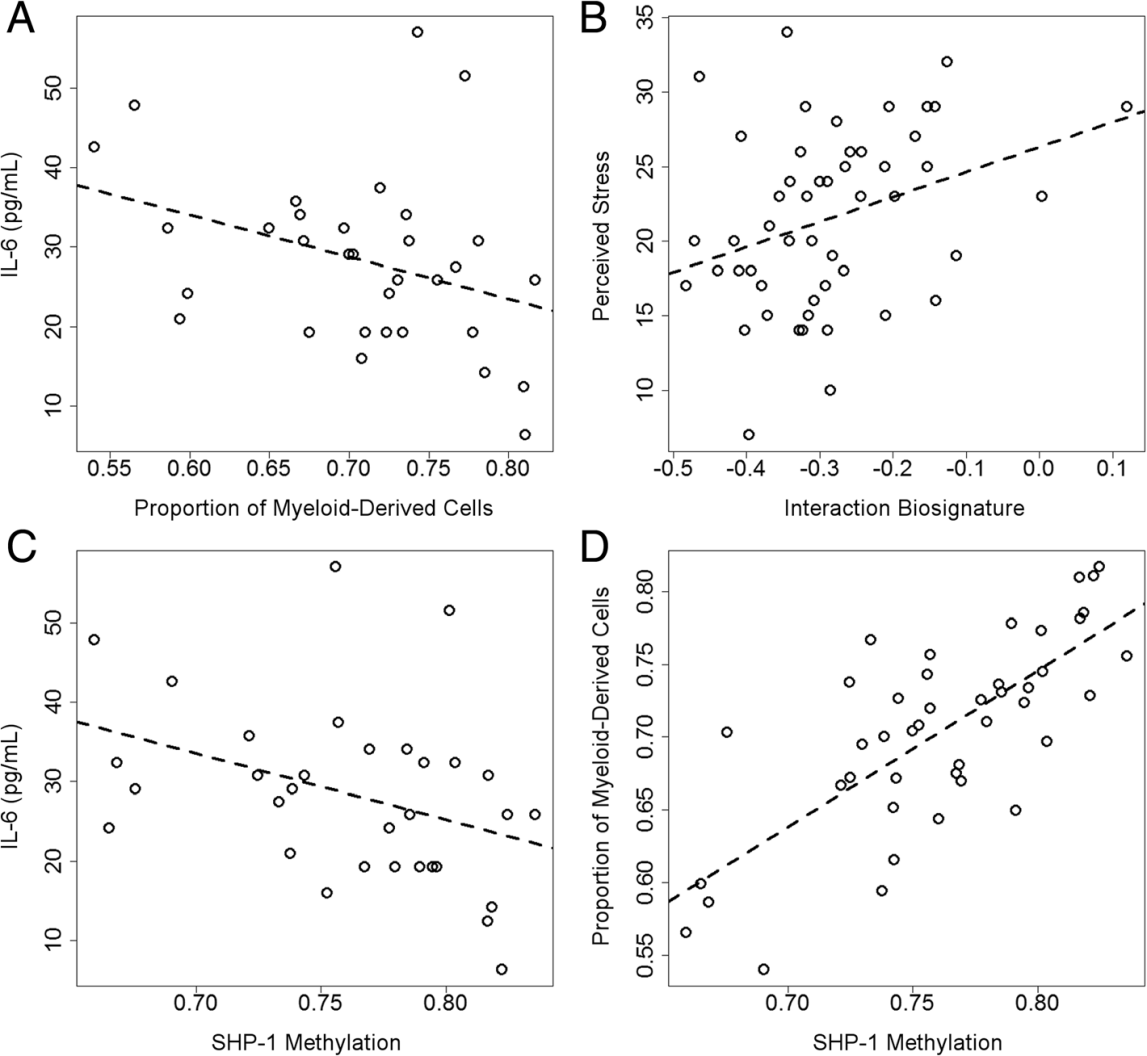
Interaction biosignature and methylation at *SHP1* (cg24437859) correlated with inflammatory markers and stress measures in the PPD cohort (trimester >1). **a** Levels of IL-6, an inflammatory marker, correlated with myeloid-derived cell proportion (*P* = 0.054). **b** The interaction biosignature correlated with the perceived stress metric (*P* = 0.019). *SHP1* methylation correlated with both **c** IL-6 levels (*P* = 0.035) and **d** myeloid-derived cell proportion (*P* = 1.4 × 10^−8^)

We reasoned that the biological underpinnings of our *SKA2* interaction biosignature may be related to variation in peripheral immune cells, as inflammation may be influenced by state factors like stress. The predicted proportion of granulocyte and monocyte content (myeloid-derived cells) showed a negative association with the interaction biosignature across all data sets (Fig. 2a–d), with significant correlations in GenRED Offspring blood (rho = −0.76, *P* = 2.7 × 10^−4^), PPD cohort blood (rho = −0.29, *P* = 0.034), and GTP blood (rho = −0.57, *P* = 2.4 × 10^−7^) and a non-significant association in GenRED Offspring saliva (rho = −0.39, *P* = 0.092). Substituting the proportion of myeloid-derived cells for the interaction biosignature in the prediction model yielded comparable predictions of SI (Additional file 4: Figure S3; Table 4) in GenRED Offspring saliva (AUC = 0.79; 95 % CI 0.56–1; *P* = 0.28), PPD cohort blood, (AUC = 0.83; 95 % CI 0.61–1; *P* = 0.003), and GTP subset (AUC = 0.73; 95 % CI 0.55–0.9; *P* = 0.99). The PPD cohort (trimester >1) showed a non-significant correlation between peripheral blood interleukin-6 (IL-6) levels and the predicted myeloid-derived cell proportion (Fig. 3a; rho = −0.32, *P* = 0.054); however, there was no correlation between IL-6 and the interaction biosignature (rho = 0.05, *P* = 0.80). Increased granulocyte and monocyte counts along with altered IL-6 levels may indicate increased inflammation and imply that our interaction biosignature could be an indicator of an immune state involved in suicide etiology [22]. The PPD cohort also showed a significant correlation between perceived stress and the interaction biosignature (Fig. 3b; rho = 0.33, *P* = 0.019), suggesting that the interaction biosignature may represent a biological state of both stress and inflammation.

### Identification of probes driving the SKA2 model interaction biosignature

Each of the 72 probes comprising the interaction biosignature was tested for correlation to the first eigenvector of the PCA model across each data set to identify subsets of probes that may be driving a majority of the variation. Three probes exhibited significant correlations (*P* < 0.05) consistent across all cohorts (Additional file 4: Table S3): cg08469255 (*DDR1*), cg22029879 (*ARHGEF10*), and cg24437859 (*SHP1*). Microarray-derived DNA methylation values were validated by pyrosequencing in select loci (Additional file 4: Figure S4). Methylation at cg24437859 used in place of the interaction biosignature predicted SI in GenRED Offspring saliva with an AUC of 0.77 (95 % CI 0.54–1; *P* = 0.20) and in PPD cohort blood with an AUC of 0.84 (95 % CI 0.63–1; *P* = 0.001). Probe cg24437859 is located within the promoter of *SHP1*, which has known immune system functions, providing a plausible biological explanation for the statistical interaction with *SKA2* identified in our data. This relationship was investigated further in the PPD cohort, where plasma cytokine levels were available. CpG methylation at cg24437859 correlated with IL-6 levels (rho = −0.37, *P* = 0.035; Fig. 3c) and the predicted myeloid-derived cell proportions (rho = 0.74, *P* = 1.4 × 10^−8^; Fig. 3d) in PPD cohort blood collected during the second or third trimester.

## Discussion

In this study, we used brain, saliva, and whole-blood DNA methylation data of several cohorts to derive a biosignature of a stress state that may aid in the prediction of suicide. Using a statistically oriented approach that analyzed cross-tissue epigenetic reprogramming by cortisol and interaction with the previous reported *SKA2* suicide biomarker, we generated an epigenetic biosignature. To assess the effects of cortisol on DNA methylation patterns, we performed a pathway enrichment analysis of genes with methylation significantly associated with AUC weekday cortisol in the GenRED Offspring samples, which revealed an enrichment of neural developmental pathways. This data is consistent with the notion that there are a number of genes that regulate both cortisol and neural development. Early life stress, for example, is known to affect both brain development and HPA axis function later in life. Several recent studies in mice reported impaired neurogenesis and cognition with early life stress [23], as well as altered CpG methylation in *NR3C1*, the gene encoding the glucocorticoid receptor [24]. Similar adverse effects have been observed in humans with exposure to early life stress [25, 26]. This link was further bolstered by an overrepresentation analysis that showed an enrichment of AUC weekday cortisol-associated genes in GenRED Offspring blood and saliva with suicide-associated genes in prefrontal neurons as well as previously identified genes associated with cortisol stress reactivity in blood [20], indicating that there are consistent cross-tissue DNA methylation changes with cortisol dysregulation and a behavioral outcome such as suicide. Our results are consistent with a model whereby suicide-associated HPA axis dysregulation causes an overproduction of circulating cortisol, which causes DNA methylation changes in various tissues, resulting in behavioral changes through the actions of DNA methylation in the brain, while leaving measurable marks in the periphery that enable the biomarker-based prediction of suicidal ideation and behaviors.

We present a biosignature that is representative of probes with a significant interaction with both *SKA2* genotype and methylation in prefrontal neurons and is predictive of suicidal ideation in three cohorts. Using this biosignature in interaction with *SKA2* can replace the stress questionnaire metrics used as interactive covariates in our original suicide prediction model. We used Monte Carlo-based testing for significance by generating a similar PCA eigenvector from randomly selected sets of 72 probes. Randomly selected probes yielded predictions inferior to that of the biosignature in almost all bootstraps, suggesting that improved model prediction accuracy is not due to the underlying data structure. The biosignature performance did not reach significance by this method in saliva, possibly due to the confounding influence of buccal-derived cell types influencing the variation generated at biosignature loci. This interaction biosignature showed correlation with percent granulocyte and monocyte (both myeloid lineage-derived cells) content in all peripheral tissue data sets, suggesting a possible immune modulation associated with the methylation at these 72 probes. Both the interaction biosignature and myeloid-derived cell levels also correlated with serum interleukin-6 (IL-6) in the PPD cohort, further suggesting that a pro-inflammatory immune modulation is interacting with *SKA2* methylation to mediate suicidal behavior. Consistent with our findings, increased pro-inflammatory cytokines, including IL-6, have been observed in the CSF of suicide attempters [27] and in the prefrontal cortex of teenage suicide victims [28, 29]. Additionally, PTSD is known to show increased levels of C-reactive protein and IL-6, which are both signs of increased inflammation [30–35]. Biological changes, such as inflammation and immune system modulation, are known to be associated with suicide and related mood disorders. For example, lymphocytes are known to play a role in HPA axis dysfunction and have been used to assess many different psychiatric disorders, including depression and suicide [36, 37]. Suicidal behavior is known to be associated with an inflammatory state which, if measured, may improve the prediction of such behavior. Substituting the percentage of myeloid-derived cells for the interaction biosignature in the prediction model was successful in predicting suicidal ideation in all of the cohorts, suggesting that this interaction biosignature may be indicative of a biological state that interacts with the trait of *SKA2* genotype and methylation to influence behavior.

In further efforts to reduce these 72 probes to a smaller number that would help us better understand the biology and facilitate practical implementation, we assessed the genes displaying epigenetic variation most closely mimicking the PCA first eigenvector. We discovered that there were three probes within the 72-probe interaction biosignature with significant correlations to the interaction biosignature in all cohorts: cg22029879, cg08469255, and cg24437859. *ARHGEF10* (rho guanine nucleotide exchange factor 10; cg22029879) was identified as one of the 21 genes located on chromosome 8p, a region that is thought to contribute to neuropsychiatric disorders, including depression [38]. Although little evidence exists tying *ARHGEF10* to suicide etiology, this locus may be worthy of further investigation due to its consistent association with the interaction biosignature across all data sets. *DDR1* (discoidin domain receptor tyrosine kinase 1; cg08469255) is primarily involved in cell adhesion and extracellular matrix remodeling but also has known roles in immune and inflammatory pathways. *DDR1* was shown in a cell culture model to induce the expression of cyclooxygenase (*COX2*), which is involved in the synthesis of prostaglandins and has a known role in inflammation [39]. *COX2* also activates the NF-κB pathway, which is involved in inflammatory pathways and cytokine production [39] and has been shown as a downstream target of *DDR1* to cause infiltrating macrophages to produce chemokines. Additionally, *DDR1* was also shown in a mouse model of kidney obstruction to mediate the development of inflammation and fibrosis following kidney injury [40]. Given this evidence, *DDR1* methylation could account for the correlation of myeloid-derived cell content with the interaction biosignature and could also represent a target for further investigation.

*SHP1* (protein tyrosine phosphatase, non-receptor type 6; cg24437859) has been implicated in modulating neutrophil recruitment to inflamed tissues through modulation of the phosphoinositol pathway [41, 42] and has been shown to play an inhibitory role in cytokine-induced activation of the HPA axis through the JAK-STAT pathway [43]. Furthermore, *SHP1* methylation correlated with IL-6 in the PPD cohort as well as the myeloid-derived cell proportion in all cohorts, altogether demonstrating biological evidence for the statistical interaction with HPA axis relevant *SKA2* identified in our data. Critically, IL-6 contributes to hematopoietic stem cell fate decisions and helps to differentiate myeloid from non-myeloid cells [42, 44]. As such, the possibility remains that epigenetic variation in genes like *SHP1* may be important not only for differentiation of hematopoietic stem cells into myeloid cells but for regulation of pro-inflammatory cytokines and may moderate the influence of pro-inflammatory cytokines on HPA axis activity. This supposition is supported by data demonstrating that *SKA2* interaction biosignature data interacted with CTQ scores to predict HPA axis responsivity in two stress challenges from multiple data sets. The relationship between the interaction biosignature signals and HPA axis sensitivity is very similar to previously reported findings related to the influence of *SKA2* DNA methylation on HPA axis activity from the same cohorts [8, 9].

In our independent validation in the GTP cohort (*N* = 376), we observed that our interaction biosignature model was only able to predict suicidal ideation in a smaller subset of drug-naïve individuals without a PTSD diagnosis (*N* = 115) but was unsuccessful predicting in the full GTP sample. Several recent papers on the usefulness of *SKA2* in predicting PTSD have observed a confounding effect of marijuana use on *SKA2* methylation, which potentially inhibits the accurate prediction of suicidal behaviors [8, 9]. Kaminsky et al. observed a significant interaction between substance abuse and *SKA2* methylation in predicting suicidal behavior in several cohorts, and Boks et al. observed altered *SKA2* methylation with smoking, alcohol consumption, and use of medications [8, 9]. Based on these findings, it is reasonable to assume that without taking into account substance abuse, using *SKA2* methylation to predict suicidal behaviors could produce inaccurate results. Along with substance abuse, trauma exposure has recently been shown to influence *SKA2* methylation [8, 9, 12]. Boks et al. showed that development of PTSD symptoms was associated with longitudinal decreases in *SKA2* methylation after military deployment, which is the opposite of the positive association between *SKA2* methylation and suicide risk [4, 8]. Furthermore, Sadeh et al. observed a positive association between genotype-adjusted *SKA2* methylation and PTSD symptom severity in one military cohort, while this PTSD association was not replicated in a second military cohort [13], adding to the complexity that is the interaction between *SKA2* methylation, PTSD, and suicidal behavior [12]. One biological explanation for this interaction is altered HPA axis function in PTSD, as shown by increased clearance of dexamethasone in the DST [45]. Kaminsky et al. recently observed decreased day 2 cortisol in the DST in subjects diagnosed with PTSD but not suicidal behavior, indicating increased HPA axis sensitivity [9]. In another study, van Zuiden et al. observed a longitudinal increase in sensitivity to dexamethasone in T cells collected from a Dutch military cohort pre- and post-deployment [46]. Another biological explanation for the *SKA2* methylation-PTSD relationship is that PTSD is associated with increased inflammation, which has been observed in many studies showing increased levels of C-reactive protein and IL-6 in the blood of both military and non-military cohorts [30–35]. The relationship between *SKA2* methylation and PTSD should be studied further to better understand the impact on suicidal behaviors.

This study has many limitations. Sodium bisulfite modification cannot distinguish between 5-methyl cytosine (5-MC) and 5-hydroxy methyl cytosine (5-HMC) levels. Like 5-MC, 5-HMC can vary in the brain in response to stress [47, 48] and has been identified in various psychiatric disorders [49, 50]. Brain tissue analyses have the potential to be confounded by postmortem factors such as the method and timing of tissue preservation. Psychiatric diseases can often be co-morbid with other illnesses such as cancer and heart disease, among others [51, 52]. Despite the implication that inflammatory factors may be interacting with SKA2, we did not assess for the health status of the study subjects and any potential impact this might have on our results. This study is limited by using small samples that are not representative of the general population and are biased towards controls due to a low ratio of cases to controls and only validated findings in a single independent sample in which suicidal behavior is only predicted in small subsets. Ideally, these findings would be further validated in a large sample that is more representative of the general population to prove its usefulness in prediction of suicidal behavior.

## Conclusions

We present a biosignature that predicts suicide consistently across multiple, highly variable data sets, specifically youth at high risk for depression, pregnant women at high risk for PPD, and middle-aged urban population with high incidence of trauma and PTSD. This biosignature is cross-tissue in that it predicts suicidal behavior in both blood and saliva samples and is based on probes that are associated with suicide in prefrontal neurons. To our knowledge, this is the first prediction model to date that works in both blood and saliva and the first suicide prediction model to use only DNA methylation to predict suicidal behavior. Additionally, correlations of the interaction biosignature with myeloid proportion and stress metrics may indicate a fuller integration of suicide etiology into the existing *SKA2* suicide prediction model. Finally, this biosignature allows us to predict suicidal behavior without using a stress questionnaire or assessment. Although the biosignature produces lower prediction AUCs than the stress questionnaires, it represents a single measure that allows us to predict suicidal behavior across all data sets, providing consistency and better allowing for comparison across populations. Ultimately, this work will add to the development of early diagnostics tests that may aid in the early identification and prevention of suicide.

## Methods

### Human samples

Peripheral blood and saliva samples were obtained from separate individuals in the GenRED Offspring cohort from Johns Hopkins [4, 53–56]. Postmortem prefrontal cortex neurons (cases, *N* = 22; controls, *N* = 23) were obtained as previously described [4], data available at NCBI Gene Expression Omnibus (GEO) accession GSE41826. Peripheral blood samples (cases, *N* = 8; controls, *N* = 43) were obtained from consenting individuals in a Johns Hopkins prospective cohort of pregnant women (PPD cohort), as previously described [57], data available from GEO accession GSE44132. Data from individuals in the Grady Trauma Project (cases, *N* = 63; controls, *N* = 313) were downloaded from the NCBI GEO accession GSE72680 [53–56]. Data on TRIER social stress test cohort (*N* = 85) was downloaded from GEO accession GSE77445 [20]. All cohorts used in model generation and validation are described in detail in Additional file 4: Table S1.

### DNA methylation analysis

Study data was derived from genome-wide DNA methylation data using the Infinium HumanMethylation450 BeadChip Array (Illumina Inc., San Diego, CA). DNA methylation profiles for GenRED Offspring cohort blood (cases, *N* = 4; controls, *N* = 14) and saliva (cases, *N* = 5; controls, *N* = 15), respectively, were generated as described below.

### Infinium chip assay

Bisulfite-converted DNA was analyzed using Illumina’s Infinium HM450 BeadChip Kit (WG-314-1001) by following the manufacturer’s protocol. The BeadChip contains 485,577 CpG loci in human genome. Briefly, 4 μl of bisulfite-converted DNA was added to a 0.8-ml 96-well storage plate (Thermo Scientific), denatured in 0.014 N sodium hydroxide, neutralized, and amplified with kit-provided reagents and buffer at 37 °C for 20–24 h. Samples were fragmented using kit-provided reagents and buffer at 37 °C for 1 h and precipitated by adding 2-propanol. Re-suspended samples were denatured in a 96-well plate heat block at 95 °C for 20 min. Twelve microliters of each sample was loaded onto a 12-sample chip, and the chips were assembled into a hybridization chamber as instructed in the manual. After incubation at 48 °C for 16–20 h, the chips were briefly washed and then assembled and placed in a fluid flow-through station for primer-extension and staining procedures. Polymer-coated chips were image processed in the Illumina’s iScan scanner.

### Data acquisition

Data were extracted using Methylation Module of GenomeStudio v1.0 Software and processed using the “minfi” and “wateRmelon” packages in R [58, 59]. Raw data was trimmed of probes failing quality assessment, followed by scale-based data correction for Illumina type I relative to type II probes. Methylated and un-methylated intensity values were then quantile normalized separately prior to the calculation of the β (beta) value based on the following definition:

β value = (signal intensity of methylation-detection probe)/(signal intensity of methylation − detection probe + signal intensity of non-methylation-detection probe + 100).

### Sodium bisulfite pyrosequencing

Microarray data was validated at select probes in the GenRED Offspring saliva cohort to corroborate array data (Additional file 4: Figure S4). Bisulfite conversion was carried out using EZ DNA Methylation-Gold Kit (Zymo Research, Irvine, CA) according to the manufacturer’s instructions on *N* = 51 subjects from the Johns Hopkins Prospective cohort. Nested PCR amplifications were performed with a standard PCR protocol in 25 μl volume reactions containing 3–4 μl of sodium bisulfite-treated DNA, 0.2 μM primers, and master mix containing Taq DNA polymerase (Sigma-Aldrich, St. Louis, MO). Primer sequences can be found in Additional file 4: Table S2. PCR amplicons were processed for pyrosequencing analysis according to the manufacturer’s standard protocol using a PyroMark Q96 MD system (QIAGEN, Germantown, MD) with Pyro Q-CpG 1.0.9 software (QIAGEN) for CpG methylation quantification. Only data passing internal quality checks for sodium bisulfite conversion efficiency, signal-to-noise ratio, and the observed versus expected match of the predicted pyrogram peak profile using reference peaks were incorporated in subsequent analyses. Data generated derive from one technical replicate.

### Blood analysis in PPD cohort

Participant blood was collected at each visit in four 10-ml EDTA tubes. Blood samples were non-fasting, and collection times were arranged at the convenience of the participant. All occurred during the working day (9:00 am to 5:00 pm). Cytokine levels have a recognized circadian rhythm but are lowest during the daytime; we were unable to control further for time of day in our analyses [60]. Samples were immediately centrifuged at 4 °C for 30 min. The plasma was then aliquoted in 2-mL microcentrifuge tubes, snap frozen on dry ice, and immediately stored in a −80 °C freezer. Cytokines were analyzed using BD Cytometric Bead Array. Plasma samples from patients were diluted 1:10 and incubated with capture beads coated with antibodies specific for IL-6. The beads were then incubated with a phycoerythrin-conjugated detection reagent containing antibodies specific to each capture bead. The capture bead + analyte + detection reagent complexes produced an individual fluorescent signal for each cytokine and were acquired on a FACSCalibur instrument. The data were analyzed using FCAP Array software. The limit of detection for IL-6 was 1.6 pg/mL. Proportions of our samples that fell below the limit of detection were as follows: IL-6, 18.9 %. Samples that fell below the limit of detection were coded “0.” All samples were run in duplicate, and the coefficient of variation between samples was <10 %. Analyses were repeated using the lowest detectable dose for those below the limit of detection, and results did not change.

### Statistical analysis

All statistical tests were performed in R (https://www.r-project.org/). Cross-reactive Illumina probes were removed from data prior to further analysis [61]. Using an Anderson-Darling test from the “nortest” package [62], all distributions of data that rejected the null hypothesis of normality were subsequently evaluated with non-parametric tests. Variance between case and control groups in each sample was assumed to be equal. All statistical tests performed were two tailed, and a *P* < 0.05 was considered significant. All statistics presented are a result of either a linear regression model or a Monte Carlo method permutation test (1000 permutations). Unless otherwise specified, ± denotes the standard deviation of the mean. Cell sub-fraction percentages were quantified for CD8 T cells, CD4 T cells, B cells, NK cells, monocytes, and granulocytes using an algorithm designed by Houseman et al. for the quantification of the cell types using DNA methylation proxies [63]. A buccal cell epigenetic profile was derived by taking the mean of *N* = 109 buccal-derived HM450 microarray profiles from a data set in GEO accession GSE25892. Incorporation of the buccal-derived profile at *N* = 500 HM450 loci into the Houseman algorithm generated training set, and incorporation of a buccal covariate was used to retrain the Houseman algorithm to quantify buccal profiles.

## Additional files

Additional file 1: Title: GenRED Offspring Blood Cortisol Associated Probes. Description: Table containing all probes nominally associated with AUC weekday cortisol in the GenRED Offspring blood cohort (N = 18) with the corresponding regression coefficient (β) and *p*-value. (XLSX 860 kb)
Additional file 2: Title: GenRED Offspring Saliva Cortisol Associated Probes. Description: Table containing all probes nominally associated with AUC weekday cortisol in the GenRED Offspring saliva cohort (N = 20) with the corresponding regression coefficient (β) and *p*-value. (XLSX 958 kb)
Additional file 3: Title: Combined GenRED Offspring Blood & Saliva Cortisol Associated Probes. Description: Table containing all probes nominally associated with AUC weekday cortisol in the combined GenRED Offspring blood and saliva cohorts (*N* = 38) with the corresponding regression coefficient (β) and *p*-value. (XLSX 1086 kb)
Additional file 4: Supplementary Data. Description: Contains supplementary results, figures, and tables. (DOCX 2399 kb)

## Abbreviations

ARHGEF10: Rho guanine nucleotide exchange factor 10
AUC: Area under the curve
CNS: Central nervous system
CpG: Cytosine-guanine dinucleotide
CTQ: Childhood Trauma Questionnaire
DDR1: Discoidin domain receptor tyrosine kinase 1
DST: Dexamethasone suppression test
GenRED: Genetics of Recurrent Early-Onset Depression
GEO: NCBI Gene Expression Omnibus
GR: Glucocorticoid receptor
GTP: Grady Trauma Project
HM450: Infinium HumanMethylation450 BeadChip array
HPA axis: Hypothalamic-pituitary-adrenal axis
IL-6: Interleukin 6
PCA: Principal component analysis
PTSD: Post-traumatic stress disorder
SA: Suicidal attempt
SCARED: Self-Report for Childhood Anxiety Related Disorders
SHP1: Protein tyrosine phosphatase, non-receptor type 6
SI: Suicidal ideation
SKA2: Spindle- and kinetochore-associated protein 2
SNP: Single nucleotide polymorphism
