# Supplementary material for: Discovery and replication of a peripheral tissue DNA methylation biosignature to augment a suicide prediction model
Source: Clin Epigenetics. 2016 Nov 3;8:113. doi: 10.1186/s13148-016-0279-1 (PMC5093988; doi:10.1186/s13148-016-0279-1)
Supplement: Additional file 4: — Supplementary Data. Description: Contains supplementary results, figures, and tables. (DOCX 2399 kb) [file 13148_2016_279_MOESM4_ESM.docx]

**Result S1.** Monte Carlo permutation was performed at each selection step of the algorithm for discovering the interaction proxy.

The first cutoff of P < 0.005 in prefrontal cortex neuron probes resulted in 669 probes. Permutation of this cutoff value was performed ×1000 by selecting 669 random probes from the list of prefrontal cortex neuron probes, then selecting from these probes those with an AUC prediction of suicide attempt in the GenRED Offspring blood cohort above the top 25% quantile. The resulting list of probes was used to build a principal component analysis (PCA) model in the GenRED Offspring blood cohort, which was then used to predict PCA models in the other data sets. The first eigenvector was used to assess suicidal ideation prediction in the original prediction model, replacing the stress measure with the PCA first eigenvector. This permutation found the algorithm to be significant or nearly significant in all cohorts assessed: GenRED Offspring blood (P = 0.186), GenRED Offspring Saliva (P = 0.147), PPD blood (P = 0.018), and GTP subset (P = 0.162).

The second cutoff of selecting AUC prediction greater than the top 25% quantile, which resulted in 72 probes, was permuted by selecting 72 random probes, building a PCA model in GenRED Offspring blood using these random probes, and then following the remainder of the algorithm. This permutation found this algorithm step to be significant or nearly significant in all cohorts: GenRED Offspring blood (P = 0.052), GenRED Offspring saliva (P = 0.146), PPD blood (P = 0.041), and GTP subset (P = 0.050).


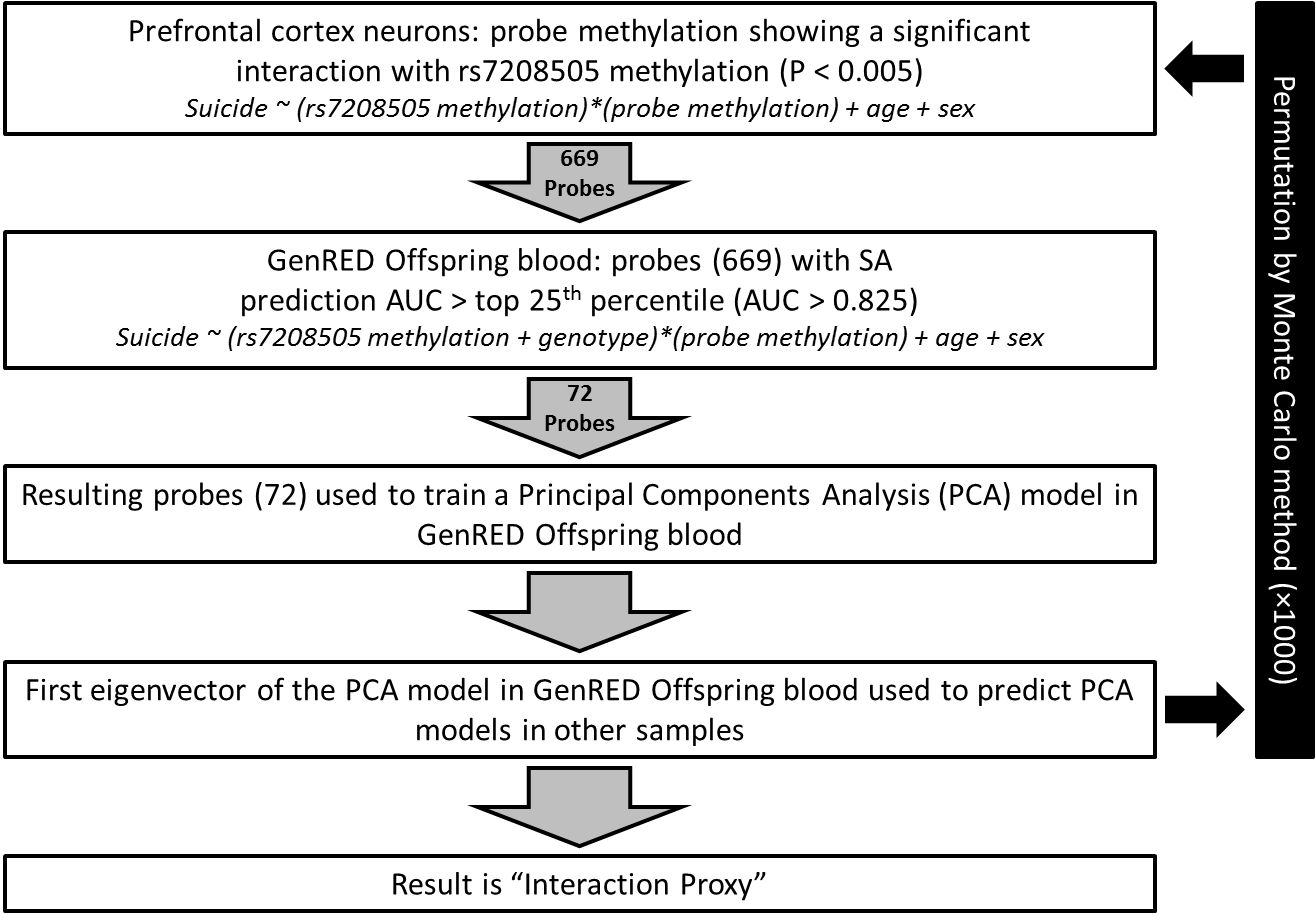


**Figure S1.** Summary of algorithm identifying Interaction Proxy.

**
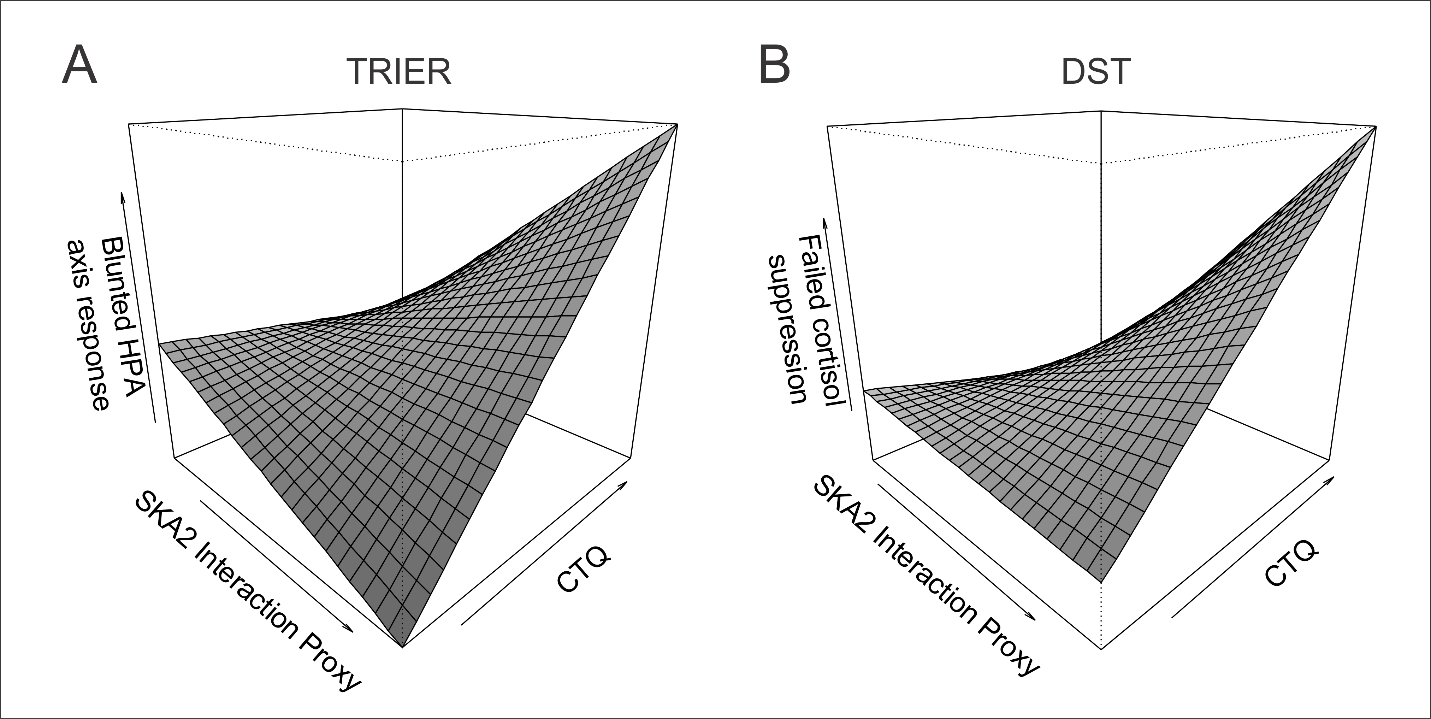
**

**Figure S2. HPA axis model depiction**

Three dimensional simulations of significant models demonstrating the interaction of SKA2 interaction proxy Eigen vector 1 with CTQ scores to influences cortisol suppression in the GTP cohort (A) and the AUC cortisol following the TRIER social stress test (B). Data for the GTP cohort derive from drug free individuals without a diagnosis of PTSD.


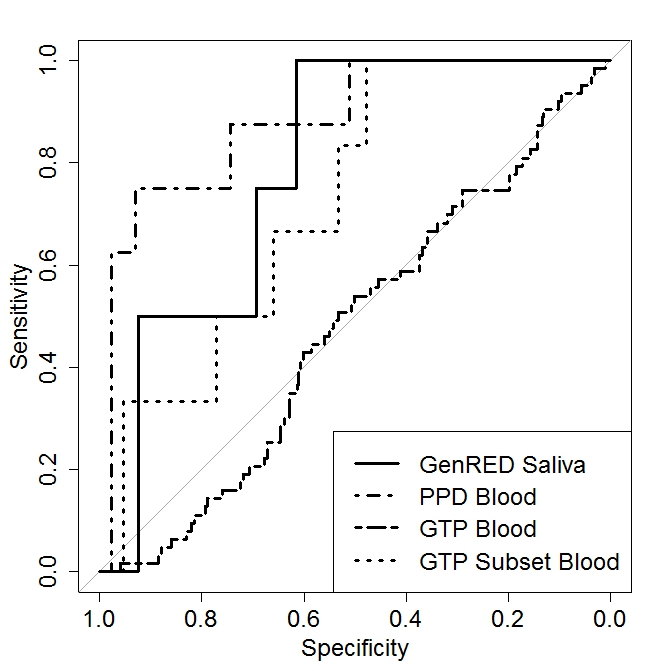


**Figure S3.** ROC curves of prediction using myeloid-derived cell proportion in place of interaction biosignature in prediction model for GenRED Offspring saliva, and PPD cohort blood, GTP blood and GTP subset blood.


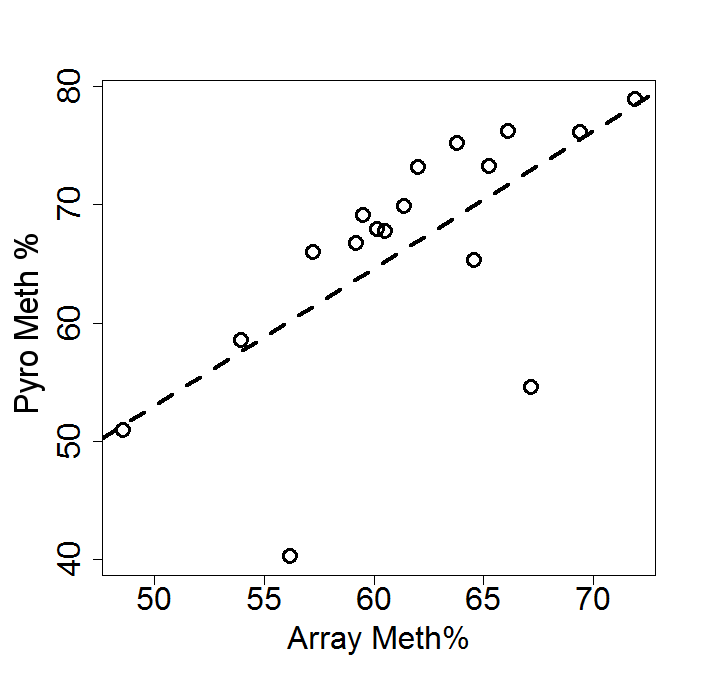


**Figure S4.** DDR1 (cg08469255) array methylation validation by sodium bisulfite pyrosequencing in GenRED Offspring saliva cohort (P = 0.0041; N = 17).

|  | **GenRED Offspring Blood** | **GenRED Offspring Saliva** | **Prospective PPD** | **Grady Trauma Project** |
| --- | --- | --- | --- | --- |
| *Diagnosis* | Depression: 50%  Bipolar: 6%  Control: 44% | Depression: 21%  Bipolar: 0%  Control: 79% | Depression: 73%  Bipolar: 27% | Depression: 41%  Bipolar: 4%  Control: 55% |
| *Sample size* | 18 | 20 | 52 | 391 |
| *Cell type* | White blood cell | White blood cell, buccal cell | White blood cell | White blood cell |
| *Ethnicity* | Cauc: 95 %  AA: 5% | Cauc: 100% | Cauc: 70%  AA: 24%  Hisp: 2%  Asian: 4% | AA: 100% |
| *rs7208505 genotype distribution* | T/T: 16.7%  C/T: 50.0%  C/C: 33.3% | T/T: 36.8%  C/T: 42.1%  C/C: 21.1% | T/T: 42.3%  C/T: 50.0%  C/C: 7.7% | T/T: 56.3%  C/T: 40.7%  C/C: 3.1% |
| *Suicidal behavior* | Ideation/Attempt | Ideation/Attempt | Ideation | Ideation/Attempt |
| *Traumatized* | 22% | 16% | 13% | 42% |
| *Prospective* | No | No | Yes | No |

**Table S1.** Sample overview.

| **Locus** | **Primer** | **Sequence** |
| --- | --- | --- |
| cg24437859 | cg24437859_Fo | GAGGTATAGAGTAGGGTATTG |
|  | cg24437859_Ro2 | CAAATCTTATTTCCTCCAAAACC |
|  | cg24437859_Fib2 | /5Biosg/TAGGGTATTGTGGTTTATTTTAGGTT |
|  | cg24437859_Ri | ATCACCTATCTCTATAAACTCCTC |
|  | cg24437859_seqR1 | AAAACAATACCTACCCTAACAA |
| cg22029879 | cg22029879_Fo | TTTAGATTTAGTTGGGATTAGGTG |
|  | cg22029879_Ro | CTATAATCCCATATACTTTTCCTAAT |
|  | cg22029879_Fib | /5Biosg/TATTAGTTTGAGTGTGAGTTAGATGTTA |
|  | cg22029879_Ri | AAACCTAAAACTAATACCTAATCCCC |
|  | cg22029879_seqR1 | TTATTTCACCCTAAATAACTCTAA |
| cg08469255 | cg08469255_Fo | GTTGTTTGTTAGTAGGTTTTTTAGT |
|  | cg08469255_Ro | TACTTTTTCCCCACTCAACAC |
|  | cg08469255_Fib | /5Biosg/GGGTTTTTGAATTAGTGTTTATGTGTG |
|  | cg08469255_Ri | TCTCCACACCCCCACACCTA |
|  | cg08469255_seqR1 | CTCACCACCTAAACAATATCA |

**Table S2.** Pyrosequencing assay primer sequences.

|  | *GenRED Offspring, blood* | | *GenRED Offspring, saliva* | | *Prospective PPD, blood* | | *GTP subset, blood* | |
| --- | --- | --- | --- | --- | --- | --- | --- | --- |
| **Probe** | **Rho** | **P-value** | **Rho** | **P-value** | **Rho** | **P-value** | **Rho** | **P-value** |
| cg08469255 | -0.54 | 0.021 | -0.46 | 0.047 | -0.28 | 0.046 | -0.42 | 3.4×10^-4^ |
| cg22029879 | -0.5 | 0.036 | -0.96 | 1.1×10^-10^ | -0.55 | 3.2×10^-5^ | -0.75 | < 2.2×10^-16^ |
| cg24437859 | -0.69 | 0.002 | 0.67 | 0.002 | -0.49 | 2.0×10^-4^ | -0.46 | 6.7×10^-5^ |

**Table S3.** Probes with significant correlations to the interaction biosignature across all data sets.
